# Isolated Penile Fournier’s gangrene: A case report and literature review

**DOI:** 10.1016/j.ijscr.2019.08.012

**Published:** 2019-08-17

**Authors:** Mohamad Moussa, Mohamed Abou Chakra

**Affiliations:** a Head of Urology Department, Zahra Hospital, University Medical Center, Beirut, Lebanon; b Faculty of Medical Sciences, Department of Urology, Lebanese University, Beirut, Lebanon

**Keywords:** Fournier’s gangrene, Penile, Isolated, Case report

## Abstract

**Background:**

Fournier’s gangrene(FG) is a rare, necrotizing fasciitis of the external genitalia, perineal or perianal regions. Penile FG is a very rare clinical entity. It is reported in only a few cases in the literature.

**Case description:**

We reported an unusual case of isolated penile FG of a 58-year-old man with uncontrolled diabetes. Our patient presented for blackish discoloration and painful swelling of the penis associated with high-grade fever. The patient was treated with immediate surgical debridement after he received broad-spectrum antibiotics. When the wound exhibited healthy bed, we used an unexpanded, meshed, split-thickness skin graft for penile reconstruction. The recovery phase was uneventful with satisfactory cosmetic results.

**Discussion:**

FG is a rare necrotizing infection of the perineum and genital region with a high mortality rate that can reach up to 50% of cases. It is frequently due to polymicrobial infection. The diagnosis is often made clinically, although radiologic studies can be helpful to define the extent of the disease. The clinical features of FG include sudden pain and swelling in the scrotum or wound discharge. Isolated penile FG are documented in few cases where the corpora cavernosa are usually spared. Its presence are related to patients sexual habits or traumatic insult. Mainstays of treatment include rapid and aggressive surgical debridement of necrotized tissue, broad-spectrum antibiotic and early resuscitation.

**Conclusion:**

Only a few cases of penile FG have been reported. Early debridement of all necrotic tissue is the golden rule.

## Highlights

• Penile Fournier’s gangrene is very rare clinical entity, which is also known wet gangrene of the penis.
• Penile Fournier’s gangrene is reported in only few reports in the literature.
• Fournier’s gangrene is usually managed using a multimodal approach.
• Early and aggressive surgical debridement is the standard initial treatment.
• Penile reconstruction, using scrotal flap or skin graft results in satisfactory functional and cosmetic outcomes.

## 1 Introduction

Fournier’s gangrene(FG) is uncommon, necrotizing fasciitis that involves the genital, perineal, and perianal regions, commonly affect men but can occur in women [1,2]. FG was first described in 1883 by JA Fournier, a French venereologist who described 5cases of penis and scrotum gangrene without an obvious cause. FG is a rapidly progressing and potentially fatal soft-tissue synergistic infection. This infection is usually caused by a mixture of aerobic and anaerobic microorganisms. The most commonly isolated microorganisms are *E. coli*, *Bacteroides* and *Streptococcus*. The mortality rate remains high and can reach up to 50% of the cases [1]. Predisposing factors are diabetes, alcohol abuse, extremes of age, malignancy, chronic steroid use, cytotoxic drugs, lymphoproliferative diseases, malnutrition, and HIV infection. The clinical features of FG are variables, it includes fever, sudden pain and swelling in the scrotum, purulence or wound discharge [2]. The diagnosis of FG is primarily clinical [1,2].

Early recognition of the pathology, fluid-electrolyte resuscitation, and aggressive surgical debridement are the mainstay of the management of FG.

Isolated FG of the penis is extremely a rarely entity. There are only a few single case reports of isolated penile FGs gangrene in the literature [3].

This work has been reported in accordance with the SCARE criteria [4].

## 2 Case report

A 58 years old male patient, with poorly controlled diabetes mellitus type 2 presented to the outpatient clinic for 4 days history of redness, blackish discoloration of the penis, painful swelling of penile shaft associated with high grade fever of 40 °C and purulent discharge from the penis. The patient denied any recent sexually transmitted disease, genitourinary trauma, urethral instrumentation. He is non-smoker and non-alcoholic. He had no sexual intercourse during the last few months. His past medical history was unremarkable except for poorly controlled diabetes (last HBA1C of 13% (normal: 4%–5.5%).

**Fig. 1:**
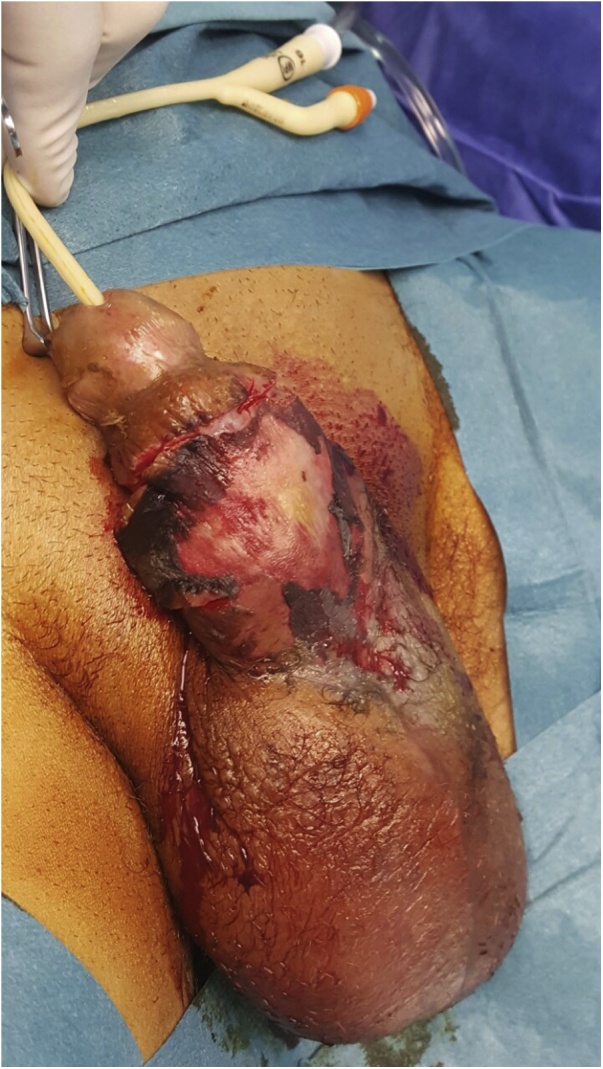
Preoperative image showed penile shaft edema, without skin breaks, normal glans and blackish discoloration of the penis.

On admission, his temperature was 40 °C and the vital signs were stable. Physical examination revealed penile edema, severe tenderness of the penile shaft with no skin breaks, normal glans, blackish discoloration of the penis (Fig. 1). The testicular, digital rectal examinations were normal. No dysuria or frequency or hematuria. He also reported chills and nausea.

Laboratory examination revealed WBC of 22000/mm3 with left shift, CRP of 240 mg/L, random blood sugar 400 mg/dl. Urine analysis showed 1–2 WBC per high power field. Blood urea & serum creatinine were within the normal limits. Purulent material discharge from penis was sent for culture. HIV test and STD panel were negative. Blood, urine, and pus cultures were obtained.

**Fig. 2:**
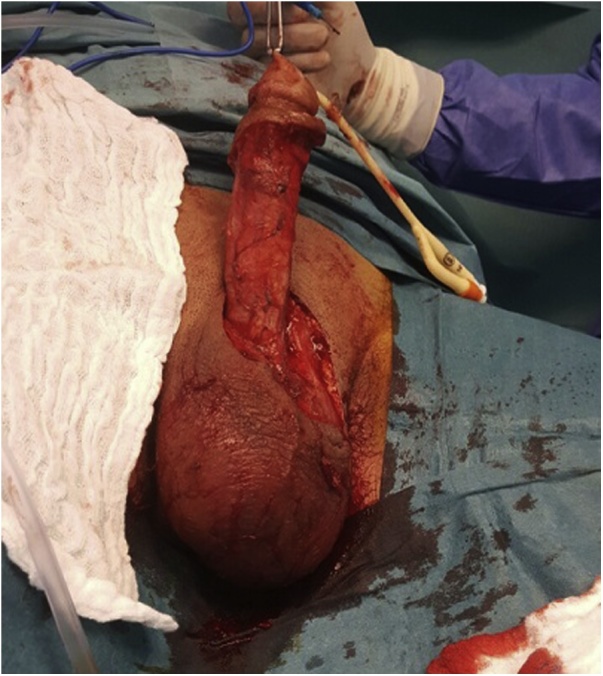
Intraoperative image demonstrating penile debridement after degloving of the penis.

The patient was started on broad-spectrum antibiotics (Ertapenem &Vancomycin) and fluid resuscitation was initiated. Urgent surgical intervention under general anesthesia was done. Before the operation, an 18-Fr Foley catheter was inserted. After degloving of the penis, it was noticed that there was a necrosis of the tissue below the skin on the ventral and dorsal aspect of the penis involving penile dartos layer up to the corpora spongiosa. Adequate debridement with excision of all necrotic tissue was done (Fig. 2). Necrotic tissue was debrided to bleeding edges. Tissue was send for culture and histopathological examination.

**Fig. 3:**
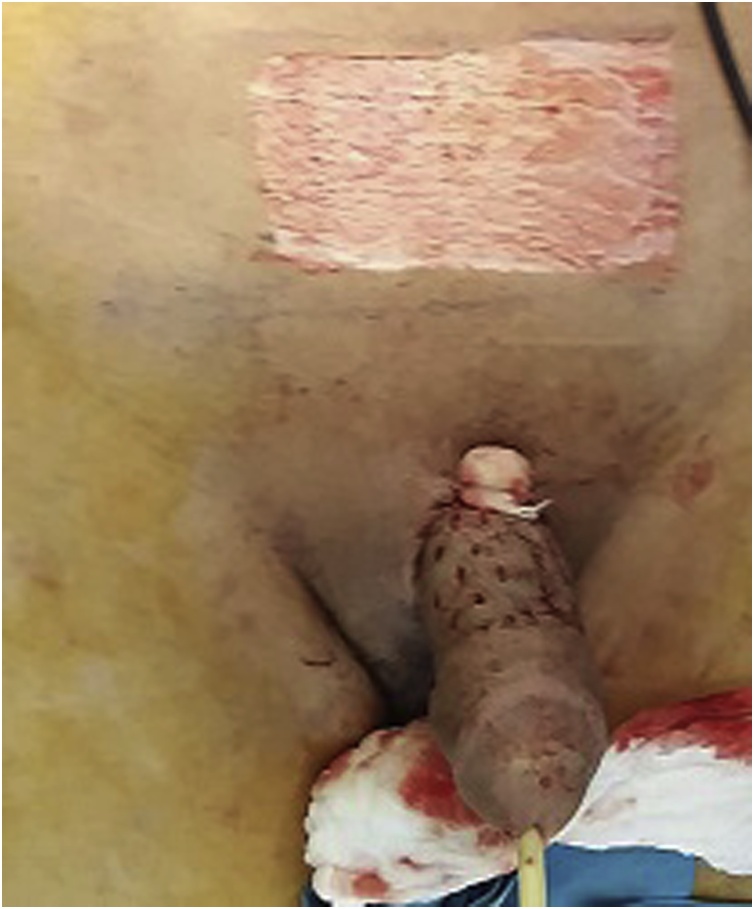
Penile reconstruction with a meshed unexpanded split-thickness skin graft.

**Fig. 4:**
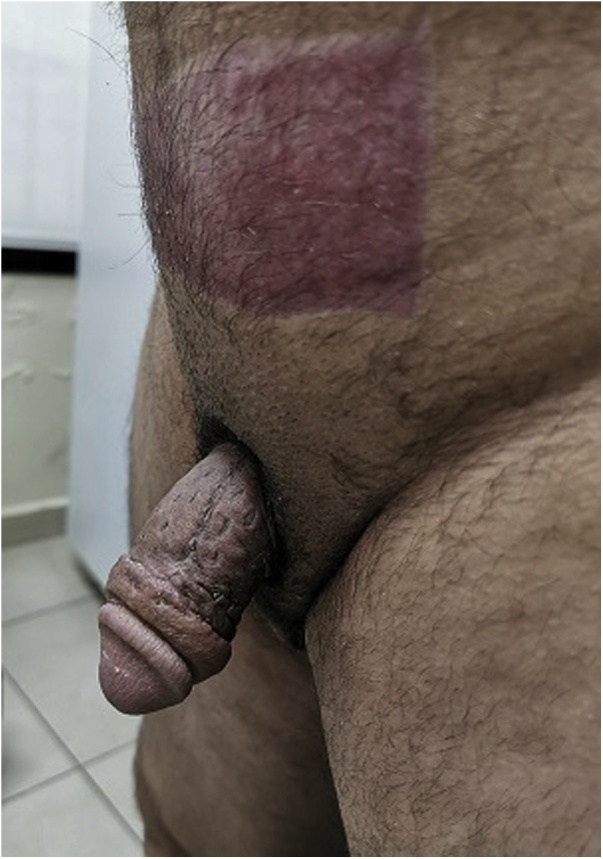
Penile form after 4 weeks of skin graft.

Postoperatively, the patient remains stable with no fever or chills. Laboratory studies improved, leukocyte count and CRP decreased. The culture of the pus materials revealed *S. aureus* and *E. coli*, the patient was completed a further 3 weeks of antibiotic according to the sensitivity test (Ertapenem 1 g once daily). Blood and urine culture revealed no growth. Regular dressing was done three times daily. After 10 days, the wound bed was granulated and healthy. An unexpanded, meshed, split-thickness skin graft was placed on the ventral and dorsal aspect of the penis (Fig. 3). The patient was discharged on the 18th postoperative day. He was seen in the outpatient clinic 3 weeks after discharge and he was markedly improved with no infection or flap necrosis was noted. A satisfied aesthetical appearance was obtained (Fig. 4). The patient provided a written consent for the publication of this clinical case.

## 3 Discussion

Fournier’s gangrene is a synergistic necrotic fasciitis of genitalia, perineum and abdominal wall. It is a rare, rapidly progressing and potentially fatal soft tissue infection, first described by JA Fournier, who described 5cases of penis and scrotum gangrene without obvious cause [1]. FG is rarely truly idiopathic; however, recent studies indicate that there is an underlying etiology. Colorectal sources (30–50% of cases), urogenital sources (20–40% of cases), cutaneous infections (20% of cases) and local trauma are frequently identified as the cause of FG [5]. Trauma to the genital or perineal region has been reported as a possible source of infection. FG has been shown to be associated with diabetes, alcohol intake, human immunodeficiency virus (HIV) and chronic steroid abuse. Those factors decreased the host immunity and allowing a portal of entry for the microorganism into the perineum [1,2].

FG exists due to synergism between multiple aerobic and anaerobic bacteria which are able to promote a rapid multiplication and spread of the infection. The most commonly isolated aerobic microorganism are *Escherichia coli*, *Klebsiella* pneumonia, and *Staphylococcus aureus* where the most commonly isolated anaerobic microorganism is *Bacteroides fragilis* [2]. Although rare, necrotizing fasciitis due to Candida species as well as Lactobacillus has also been reported. The clinical features of FG include sudden scrotal pain and swelling associated with systemic features such as fever greater than 38 °C. Examination reveals purulent discharge and area of necrosis [1].

The diagnosis of FG is primarily based on clinical findings. Imaging studies may be useful in those cases where the presentation is unusual or to define the extent of the infection. Computed tomography (CT) has greater specificity for evaluating disease extent than does radiography or Ultrasound(US). The CT features of FG include soft-tissue thickening and inflammation, asymmetric fascial thickening, fluid collection or abscess, fat stranding around the involved structures, and subcutaneous emphysema. US finding in FG is a thickened, edematous scrotal wall and reactive hydroceles [6].

The cornerstones of management are urgent patient resuscitation, broad-spectrum antibiotic therapy, surgical debridement and reconstruction surgeries. Parenteral broad-spectrum antibiotics are required, it includes a triple therapy: third-generation cephalosporins or aminoglycosides, plus penicillin and metronidazol then adjusted according to the result of the cultures. Early surgical debridement is always recommended where necrotic tissue must be performed until the wound bed is clean [1,2,5].

Isolated FG of the penis is uncommon due the highly vascular nature of the penis [7]. There are only a few single case reports of isolated penile Fournier’s gangrene in the literature. Literature search revealed twenty-one cases (Table 1).

**Table 1:** Published reports of penile Fournier’s gangrene.

Reference	Year	Number of Case(s)	Predisposing factors
Bernstein et al. [7]	1976	3	Bite during sexual activity
Schneider et al. [8]	1986	2	Urethral stricture
Eke N et al. [9]	1999	1	Adenocarcinoma of the rectum & diabetes mellitus
So A et al [10]	2001	1	Calciphylaxis of the penis
Mouraviev VB et al. [11]	2002	1	Penile self-injection with cocaine
Tauro LF et al. [12]	2005	1	Idiopathic
Anchi T et al. [13]	2009	1	Abrasion of the penis during oral sex
Talwar A et al. [14]	2010	1	Idiopathic
Yecies T et al. [15]	2013	1	Calciphylaxis secondary to end-stage renal disease
Akbulut F et al. [16]	2014	1	Diabetes mellitus
Temiz MZ et al. [17]	2015	1	Diabetes mellitus
Obi AO et al. [3]	2016	4	Long segment penile urethral stricture, penile abrasion from oral sex, penile edema from poorly controlled congestive cardiac failure or Idiopathic
Deb PP et al. [18]	2018	1	Idiopathic
Katsimantas A et al. [19]	2018	1	Idiopathic
Our case	2019	1	Idiopathic

Our case was unusual in that only the penis was involved, without any involvement of the scrotum or abdominal wall, the only existing predisposing factor was uncontrolled diabetes mellitus. There was no detectable portal of entry. Despite the continuity of the superficial fascial planes of the penis and scrotum and the dependent position of the scrotum, we have not observed the spread of penile Fournier's gangrene to the scrotum, the reason for this is not known.

## 4 Conclusion

Fournier’s gangrene is a fulminant form of infective necrotizing fasciitis of the perineal, genital, or perianal regions. It is a urological emergency. Management of such condition is usually using a multimodal modality, which includes early resuscitation, broad-spectrum antibiotics and extensive surgical debridement with future reconstructive surgeries. Isolated FG at the penis is unusual and only a few cases are reported. More data are needed to better define optimal management strategies.

## Sources of funding

No funding.

## Ethical approval

Ethical approval is not required by our institution.

## Consent

Written informed consent was obtained from the patient for publication of this case report and accompanying images.

## Author contribution

Mohamed Abou Chakra, Mohamad Moussa: Case report design.

Mohamed Abou Chakra, Mohamad Moussa: Manuscript preparation.

Mohamed Abou Chakra, Mohamad Moussa: Followed up the patient and revised the manuscript.

Mohamed Abou Chakra, Mohamad Moussa: Approved the final manuscript.

## Registration of research studies

Not applicable, case report.

## Guarantor

Mohamed Abou chakra.

## Provenance and peer review

Not commissioned, externally peer-reviewed.

## Declaration of Competing Interest

None identified.
